# Variation in haplotypes in single cysts of assemblages C and D, but not of assemblage E of *Giardia duodenalis*

**DOI:** 10.1186/s12866-022-02581-3

**Published:** 2022-06-27

**Authors:** Floor L. Veldhuis, Rolf Nijsse, Jaap A. Wagenaar, Ger Arkesteijn, Frans N. J. Kooyman

**Affiliations:** Department Biomolecular Health Sciences, Faculty of Veterinary Medicine, Utrecht University, Utrecht, The Netherlands

**Keywords:** Parasitology, Allelic Sequence Heterozygosity, Haplotypes, *Gdh*, *dis3*, Dog

## Abstract

**Background:**

*Giardia duodenalis,* a single-celled intestinal parasite, is divided into eight assemblages (A-H), with differences in host specificity. *Giardia duodenalis* reproduces asexually and cycles between the binucleated trophozoite (4 N) and the infectious cyst with four nuclei (16 N). Interaction between the nuclei is limited. Therefore, genetic drift causes differences in genetic make-up between the non-daughter nuclei; the allelic sequence heterozygosity (ASH). The ASH is low (0.01%—0.0023%) for the related assemblages A and E, higher (0.43–0.53) for assemblage B and much higher (0.74% -0.89%) for the assemblage C and D at the root of the phylogenetic tree. The heterozygosity in assemblage F, in the same clade as assemblage A and E, was unknown. The heterozygosity in the sequences of the *gdh* and *dis3* genes was used as proxy for the ASH and whole genome amplification of single cysts followed by cloning and Sanger sequencing of *dis3* fragment could reveal the genetic variation within the cyst. The aim of the study was to determine the level of heterozygosity within pooled and single cysts of different assemblages.

**Results:**

The heterozygosity in *gdh* and *dis3* was determined in pooled cysts of the assemblages A to F. Heterozygosity in the isolates of the assemblages C (*n* = 2) and D (*n* = 1) ranged from 0.41% to 0.82% for *gdh* and *dis3* and no heterozygosity was found in the isolates of the assemblages A (*n* = 4), E (*n* = 3) and F (*n* = 3). The heterozygosity in assemblage B (*n* = 7) was intermediate (0% to 0.62%). Next, the number of haplotypes of *dis3* was determined for single cysts of assemblages C, D and E. In the assemblages C and D, two to four haplotypes were found per cyst, while in assemblage E only one haplotype was identified.

**Conclusions:**

Having high heterozygosity is characteristic for the assemblages C and D, while having a low heterozygosity is characteristic for the clade with the assemblages A, E and F.

Presence of more than 1 haplotype per cyst in assemblage C and D suggests differences between the non-daughter nuclei, in contrast to the one haplotype in assemblage E.

**Supplementary Information:**

The online version contains supplementary material available at 10.1186/s12866-022-02581-3.

## Introduction

*Giardia duodenalis* (syn. *Giardia intestinalis, Giardia lamblia*) is a single-celled binucleated parasite, infecting millions of people, and numerous mammals a year. Infections vary greatly in disease outcome, ranging from asymptomatic infections to giardiasis with severe diarrhoea, vomiting, bloating, nausea, and fatigue. In low-resource countries, infections are linked to maldigestion and malabsorption, mortality, and retardation in development (both physical as cognitive) in children [1]. *Giardia duodenalis* is a species complex that can be divided into eight assemblages (A-H). The assemblages differ, amongst other things, in host specificity and zoonotic potential. Assemblage A and B can be identified in infections in a broad range of mammals including humans; C and D in dogs/canids; E in hoofed animals; F in cats/felids; G in rats and H in marine mammals [2]. Assemblages A and B can be divided further into sub-assemblages AI, AII, AIII and BIII, BVI, respectively, leading to a more specified host specificity [2].

*G. duodenalis* is known to reproduce asexually [3] and has two life cycle stages, the trophozoite and the cyst. The trophozoite has two transcriptionally active nuclei with a total ploidy of 4 N. During encystation, the trophozoite undergoes an incomplete mitotic division, without cytokinesis, resulting in 8 N ploidy. Another division takes place within the cyst, resulting in four nuclei with a 16 N ploidy of the cyst [4]. *Giardia duodenalis* has a compact genome, ~ 11.7 Mb in size, distributed over five chromosomes [5]. Without the exchange of DNA between the non-daughter nuclei, variation between these nuclei would emerge and increase over time due to genetic drift, resulting in an increased percentage of variable nucleotides within the genome. This variation has been determined for various assemblages based on whole genome sequence data and is called the allelic sequence heterozygosity (ASH) [6]. The ASH in the assemblages varies over a wide range; with (sub)assemblages AI, AII and E showing low ASH rates: < 0.01%, 0.037% and 0.0023%, respectively, in contrast to assemblages B, C and D having a high ASH: 0.53%, 0.89% and 0.74%, respectively [5–9]. The ASH in assemblages C and D is based on single cysts, in contrast to assemblage A, B and E which have been determined based on pools of trophozoites grown in axenic culture. This latter method makes it difficult or impossible to distinguish whether the variation found is located between or within individual trophozoites.

The whole genome sequences from single cysts of assemblage C and D were performed on MiSeq platforms (illumina) with 2 × 250 bp reads. This way the number of single nucleotide polymorphisms (SNPs) could be determined, but it was not possible to determine the distribution of the SNPs over the different haplotypes, and therefore, the number of haplotypes in a single cyst could not be determined. Only two studies described the variation within single *Giardia* cysts and both studies were on cysts and/or trophozoites from human isolates [10, 11]. Both studies found variation within a single cyst or trophozoite. Aguilar et al. (2015) found after cloning, up to 14 alleles within a single *Giardia* cyst and this suggests contamination and/or artefacts. Variation was also reported within a single cyst or trophozoite of assemblage B [11]. They did not clone the fragment and therefore the number of haplotypes is not known.

Having this fundamental difference in low or high ASH should have implications for this parasite and should have impact on the evolution of the (sub)species. A high ASH resembles the situation of a heterozygote, whereas a low ASH resembles a homozygote organism.

An explanation for the low ASH in some of the assemblages is a process called diplomixis, found in parasites of assemblage A [12]. Diplomixis is the process of nuclear fusion of non-daughter nuclei within a single cyst in which genetic exchange in the form of homologous recombination takes place. Other studies found also signs of intra and inter-assemblage recombination in genome sequences [3, 13]. When looking at the phylogenetic tree based on the whole genome, assemblages A and E with a low ASH, diverge late in the evolutionary timeline [9]. The genome of assemblage F is not known, but based on a single locus (glutamate dehydrogenase, *gdh*), it groups together with assemblages A and E [14]. If having a low ASH is a derived character, assemblage F is expected to have a low ASH as well.

The aims of the present study were 1) Estimating the level of heterozygosity of several isolates from the assemblages A to F by using the variation within 2 genes as a proxy for the ASH. The variation within assemblage F is unknown, but we hypothesized that assemblage F, being in the clade with assemblages A and E, has a low ASH. If true, we hypothesized that having a low ASH is a shared, derived character of the monophyletic clade containing the assemblages A, E and F. 2) Estimating the variation and number of haplotypes within single cysts of the assemblages C and D with a high ASH by cloning and Sanger sequencing of the single copy gene *dis3*. For this, the same cysts from which the whole genome is published are used [9]. Because of the 4 N character of the binucleated *Giardia*, we hypothesized that there will be no more than four haplotypes of the *dis3* gene within a single cyst.

## Methods

### Samples and preservation of the cysts

All cysts were isolated from fecal samples from various animal species that were sent for diagnostic purposes to the Veterinary Microbiological Diagnostic Center (VMDC) (Table S1). Cysts were isolated and stored in 70% ethanol at 4 °C as described [9].

### DNA isolation of pooled cysts

A volume of cyst suspension in ethanol containing 10,000 cysts was centrifuged (14,000 g, 2 min) in 2 ml eppendorf tubes. The pellet was used for DNA isolation with the GeneJet Genomic DNA purification kit (ThermoFischer Scientific) according to [15], except for the small modification that, after adding digestion buffer, samples were frozen at -80 °C for 15 min.

### Amplification of *gdh* and *dis3* fragment from pooled cysts

The heterozygosity in *gdh* (glutamate dehydrogenase) and *dis3* (mitotic control protein) was comparable to the heterozygosity of the whole genome (ASH) [9] and were therefore used as proxy for the heterozygosity. Both fragments were amplified from genomic DNA of pooled cysts with Dreamtaq (ThermoFisher Scientific) supplemented with 0.5 mg BSA/ ml (Sigma) in nested polymerase chain reaction (PCR). All primers are given in Table S2. The amplification of *gdh* with first primer pair gdh-1 and gdh-2 started with an initial denaturation step of 95 °C for three min followed by 35 cycles (95 °C for 30 s, 57.5 °C for 30 s and 72 °C for 1 min) and ended with a final extension step of 72 °C for 7 min. The second amplification with gdh-3 and gdh-4 primers started with an initial denaturation step of 95 °C for 3 min followed by 35 cycles (95 °C for 30 s, 60 °C for 30 s and 72 °C for 1 min) and ended with a final extension step of 72 °C for 7 min.

The amplification of *dis3* with first primer pair DISF1 and DISR1 started with an initial denaturation step of 95 °C for 3 min followed by 35 cycles (95 °C for 30 s, 53 °C for 30 s and 72 °C for 3 min) and ended with a final extension step of 72 °C for 7 min. The second amplification with DIS3F2 and DISR2 primers started with an initial denaturation step of 95 °C for 3 min followed by 35 cycles (95 °C for 30 s, 50 °C for 30 s and 72 °C for 3 min) and ended with a final extension step of 72 °C for 7 min.

All PCR products were analyzed on 1% agarose gel, stained with Midori green and imaged under UV light.

### Labelling, FACS and multiple displacement amplification (MDA) of the single cysts

The labeling, isolation by FACS and MDA amplification of the single cysts from assemblage C and D from isolate 1 were described [9]. In short, the cyst suspension in ethanol was added to zirconium beads and were washed with PBS. Staining was done with detection reagent (Merifluor C/G, Meridian Bioscience), containing FITC-labelled mAb against *Giardia* and *Crytosporidium*.Stained cyst were checked for the absence of *Cryptosporidium* and cysts were diluted to 5 cysts per µl PBS. Single cysts were isolated by FACS and single cysts were amplified by MDA with REPLI-g single cell kit (Qiagen). The same MDA product from cyst 1C, 3C (assemblage C) and 2D, 4D (assemblage D) was used for whole genome sequencing of single cysts and for the present study.

Single cysts from assemblage E were labeled slightly different. The ethanol suspension containing at least 20,000 cyst was spun on Multiscreen filterplate (1.2 µM MSBVN1210, MultiscreenHTS BV) for 30 s at 100 × g. Spinning was repeated twice with 150 µl PBS and cysts were harvested in 150 µl PBS. For counting of the nuclei, the cysts were stained with FITC-labelled monoclonal antibody (mAb) against the cyst wall (Merifluor C/G, Meridian Bioscience) as described [9] and with 2-[4-(Aminoiminomethyl)phenyl]-1H-Indole-6-carboximidamide hydrochloride (DAPI). One µl DAPI (1 mg/ml) was added to 150 µl cysts suspension in PBS together with FITC-labelled mAb and cysts were stained for 30 min at room temperature in the dark. The excess of stain was removed by spinning twice on the Multiscreen plate. The number of nuclei per cysts was estimated by fluorescent microscopy (Olympus BX60) at 1000 × amplification. The sorting by FACS and MDA by REPLI-g single cell kit (Qiagen) of the cysts was as described for single cysts of assemblage C and D [9].

### S1 nuclease treatment

Prior to PCR amplification, MDA products were treated with S1 nuclease (ThermoFisher Scientific) to remove single stranded DNA. Two μl MDA product was added to the reaction buffer (4 μl 5X reaction buffer, 1 μl 10X diluted S1 nuclease in 1 × reaction buffer, 13 μl H2O) and incubated for 30 min at RT. Reaction was stopped by addition of 1.33 μl 0.5 M EDTA and heating for 10 min at 70 °C.

### Amplification and cloning of *dis3* fragment from MDA products

In order to reduce artefacts, SI nuclease treated MDA products were used as template [16, 17], amplifications were optimized for minimal number of cycles and long elongation times [18], and a high-fidelity polymerase (Phusion hotstart II with HF buffer, ThermoFisher Scientific) was used. PCR reactions contained: 2.5 ul template (100 × diluted MDA/SI nuclease treated product). The degenerated DIS3F2 primer was used for all assemblages and reverse primers C_dis3_R1100, D_dis3_R1100 and DIS3R2 were used for assemblages C, D and E, respectively (see Table S2). Cycling conditions for all reactions started with an initial denaturation step of 98 °C for 30 s followed by 25 cycles (98 °C for 10 s, 61 °C for 30 s and 72 °C for 120 s) and ended with a final extension step of 72 °C for 7 min.

The *dis3* fragments were cloned. For this, the *dis3* PCR products from individual cysts were run on 1% agarose gel and the band of interest was cut from the gel and DNA was purified from the gel with GFX PCR DNA and Gel Band purification kit (GE Healthcare) according to manufacturer’s protocol. The A ‘ overhang was created with Taq (ThermoFisher Scientific) for 20 min at 72 °C. Next, the insert was ligated into pGEM-easy vector (Promega) according to manufacturer’s protocol overnight at 4 °C. *E. coli* JM109 cells were transformed by heat shock. To increase the chance that the selected clones originated from a unique vector-insert combinations, the JM109 cells were incubated in 12 separate vials directly after transformation, grown for one hour without antibiotics and plated onto separate quarters of LB/ampicillin (100 μg/ml) plates. Each plate contained 100 μl IPTG (0.1 M) / 20 μl X-gal (50 mg/ml) for blue, white screening and plates were incubated overnight at 37 °C. White clones, originating from the 12 separate plate quarters, were picked, added to 25 µl Worm Lysis Buffer (WLB) and Proteinase K (ProtK)(19) and heated for 30 min at 56 °C followed by 15 min at 95 °C. The lysed clones were screened by PCR without further purification.

### Amplification of *dis3* fragment from clones

The WLB/ProtK lysed clones (2.5 µl) were amplified with DreamTaq polymerase (ThermoFisher Scientific) supplemented with 0.5 mg BSA/ml (Sigma) and vector specific primers T7 and M13 (Tabel S2). Amplification started with an initial denaturation step of 95 °C for three min followed by 35 cycles (95 °C for 30 s, 49 °C for 30 s and 72 °C for three min) and ended with a final extension step of 72 °C for seven min.

### Distribution of haplotypes and removal of singleton sequences

Assuming no occurrence of mutation and/or recombination in the *dis3* fragment during formation of a single cyst, at most four haplotypes of the *dis3* fragment are expected in a single cyst. The chance of missing haplotypes depends on the number of clones analyzed and was calculated with binomial test in GraphPad Prism. With 24 clones, the probability for a haplotype to be absent in all the clones is 0.1% (Table S3). This was considered acceptable and therefore, 24 clones were analyzed of each cyst. With 24 clones the chance a haplotype is recovered only once is < 1.0% (Table S3). Therefore, singletons (sequences occurring only once) were considered PCR, MDA and/or sequence artefacts and are removed from the data set before analysis.

With maximal four haplotypes, each at a frequency of 0.25, there is a limited variation in frequency for the four haplotypes (H1, H2, H3, H4), being (1.0, 0, 0, 0), (0.75, 0.25, 0, 0), (0.5, 0.5, 0, 0), (0.5, 0.25, 0.25, 0) and (0.25, 0.25, 0.25, 0.25). Probability of the observed haplotype frequency (number of clones) to be in agreement with the expected frequency of the haplotypes was calculated with Chi-square in GraphPAd Prism.

### Sequencing and sequence analysis

PCR products from pooled cysts and from clones from single cysts were treated with Exo-SAP-IT (affymetrics) and sent to Baseclear (Leiden, The Netherlands) for Sanger sequencing in both directions. Sequences were checked for double signals (ambiguous nucleotides) by SeqMan Pro (DNASTAR lasergene v16). Alignments in Muscle, as well as the translation of DNA sequences (Hexamita nuclear codes) and calculation of genetic distances were performed in MEGA-X with maximum composite likelihood model with gamma distribution. Phylogenetic trees were built in MEGA-X with maximum likelihood methods and Tamura-nei model with five discrete categories of gamma distribution and complete deletion of missing positions. The tree based on *gdh* and *dis3* from pooled cysts was evaluated by bootstrapping (500 times). In the phylogenetic analysis of the clones, identical clones were counted. Sequences occurring only once were discarded (see above) and sequences occurring in more than 2 clones were indicated as a cluster. The sequences were submitted to Genbank, accession numbers ON360020-ON360071.

## Results

### Genotyping of the isolates

**Fig. 1:**
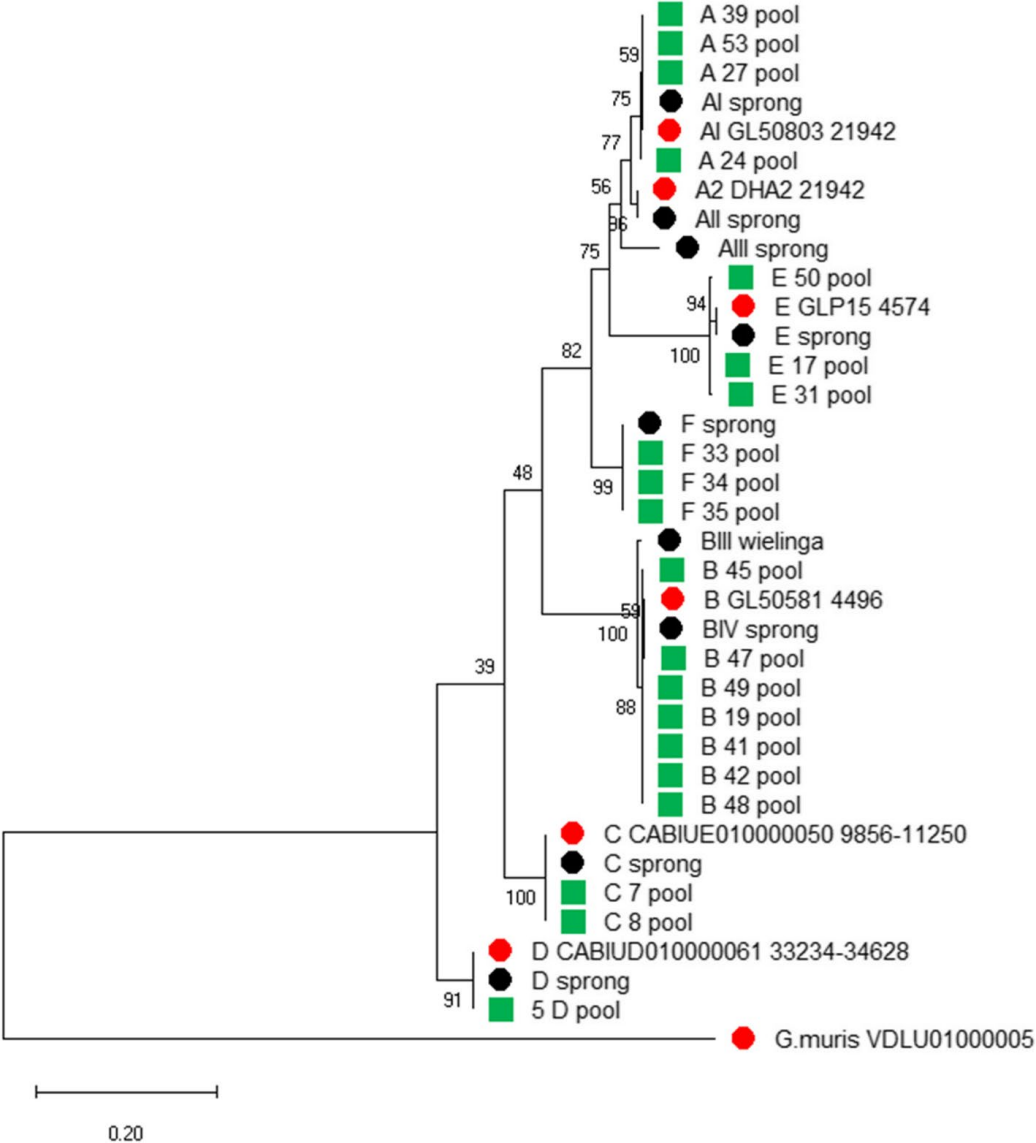
Phylogenetic analysis of *gdh* fragments amplified from pooled cysts by maximum likelihood method. Samples with the assemblage and the number of the isolate (green squares), reference sequences (black dots) and reference sequences obtained from genome sequence projects (red dots) are indicated. Bootstrap values are indicated at the branching points. Bar indicates number of nucleotide substitutions per site

The *gdh* and *dis3* PCR products obtained from pooled cysts of all the isolates were sequenced and a phylogenetic tree was constructed. A rooted tree based on the *gdh* product was constructed, together with reference sequences of all assemblages [2] and the sequences from whole genome sequencing projects of assemblages A to D, with *G. muris* as the outgroup (Fig. 1). For construction of the phylogenetic tree with the *dis3* fragments only whole genome sequences of assemblage A to E were available as reference (Figure S1). Genotyping by *gdh* classified all isolates to the same assemblage as genotyping by *dis3* and all the isolates were genotyped to the assemblages A to F (Table S1). In the rooted *gdh* tree (Fig. 1), the assemblages C and D are located near the root and the assemblages A, E and F are at the top of that tree with assemblage B as a sister-clade. In both trees, the assemblages A, E and F form a monophyletic clade.

### Ambiguous positions in *gdh* and *dis3* fragments of pooled cysts

In the aligned Sanger sequences of the *gdh* and *dis3* fragments of the isolates the ambiguous positions (double peaks) were identified (Tables 1 and 2). No ambiguous nucleotides were found in the *gdh* or *dis3* fragments of the assemblages A, E and F. Out of the seven isolates of assemblage B, four and three isolates showed ambiguous positions in the *gdh* and *dis3* fragments, respectively. All isolates from the assemblages C and D showed ambiguous positions in both the *gdh* and the *dis3* fragments. For all assemblages the ASH based on the genome sequences was reflected in the percentage heterozygosity found in the *gdh* and *dis3* fragments.

**Table 1:**
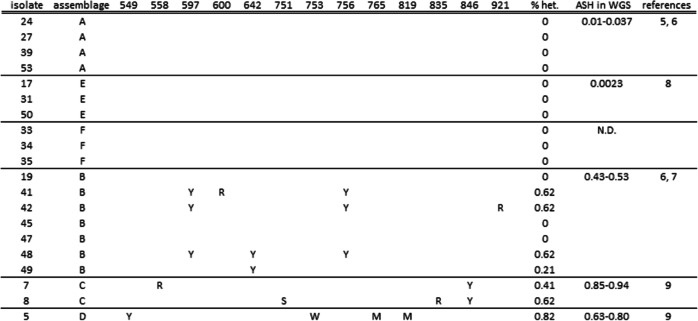
Ambiguous nucleotides in PCR product of *gdh* (487 bp) from 10,000 pooled cysts. Posistion refers to position of *gdh* in assemblage C (1350 bp)

**Table 2:**
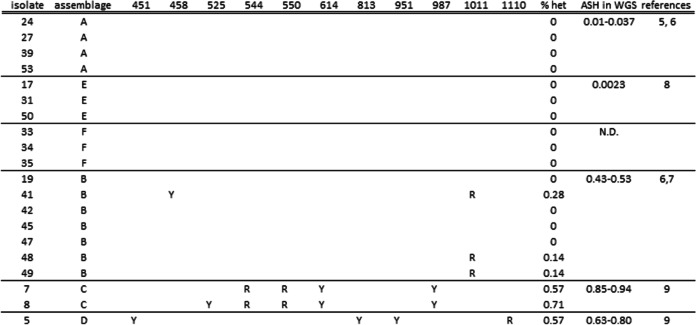
Ambiguous nucleotides in PCR product of *dis3* (702 bp) from 10,000 pooled cysts. Posistion refers to position of *dis3* in assemblage C (3369 bp)

Sequences obtained from pools of cysts without ambiguous nucleotides (as in assemblages A, E and F), demonstrated the absence of heterozygosity in *gdh* and *dis3* between and within cysts. However, ambiguous nucleotides in samples of pooled cysts of the assemblage B, C and D, can be caused by variation between cysts or by variation within single cysts. Therefore, variation within single cysts was determined in the assemblages with the highest level of heterozygosity, assemblages C and D.

### Isolation single cysts

The single cysts 1C and 3C (assemblage C) and cysts 2D and 4D (assemblage D), all from the same isolate 1, were isolated and the whole genome amplification and sequencing was performed previously [9]. The isolation and whole genome amplification of cysts from assemblage E was performed separately. The labeled cysts (*n* = 37) from assemblage E had clear nuclei and four nuclei were seen in 32 cysts (87%), three nuclei in three cysts (8%) and two nuclei in two cysts (5%). The sorting of the cysts resulted in one single peak in DAPI signal, indicating the same amount of DNA in all cysts (Figure S2).

### Heterozygosity within single cysts

We previously demonstrated genetic variation within single cysts of assemblages C and D [9], but were not able to determine the distribution of the ambiguous positions over the different haplotypes within a cyst. Therefore, the fragments with the most ambiguous positions in the pooled cysts of the isolates 5, 7 and 8 from assemblages C and D were determined (*dis3*). Next, these fragments were amplified and cloned from the same MDA product of the same cysts; cyst 1C, 3C, 2D and 4D as used in the previous study [9]. The *dis3* fragment from the MDA product of a single cyst of assemblage E, isolate 50 (cyst 50E), in which the pool did not show any ambiguous position (Tables 1 and 2) was also amplified and cloned.

The ambiguous positions in all *dis3* clones from the single cysts are given in Table S4. Clones containing insert sequences occurring only once (singletons) were considered artefacts and were removed before analysis. The number of ambiguous positions without the singleton sequences ranged from four to ten for assemblages C and D (Table 3) and no ambiguous sites for cyst 50 from assemblage E were found (not shown). The number of haplotypes per cysts varied between two and four for assemblage C and D and only one haplotype was found in cyst 50E. The deduced amino acid sequences differ in the haplotypes at four and three positions for cyst 1C and 3C, respectively and at two and four positions in cyst 2D and 4D, respectively. The probability of the observed numbers of clones per haplotype is given for the five possible haplotype distributions (Table S5).

**Table 3:**
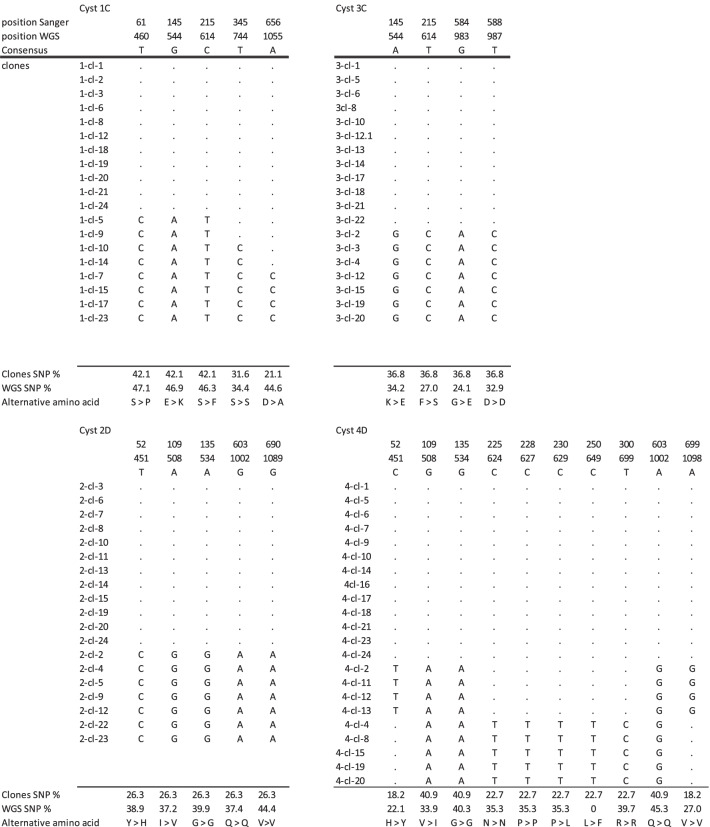
Variable sites in clones of *dis3* framets (665 nt) from single cyst. Cyst 1 and 3 from assemblage C and cyst 2 and 4 from assemblage D. Position whole genome sequence (wgs) correspond to full length *dis3* of assemblage C (3369 nt) and (3354 nt). No variable sites were detected in single cyst of assemblage E

**Fig. 2:**
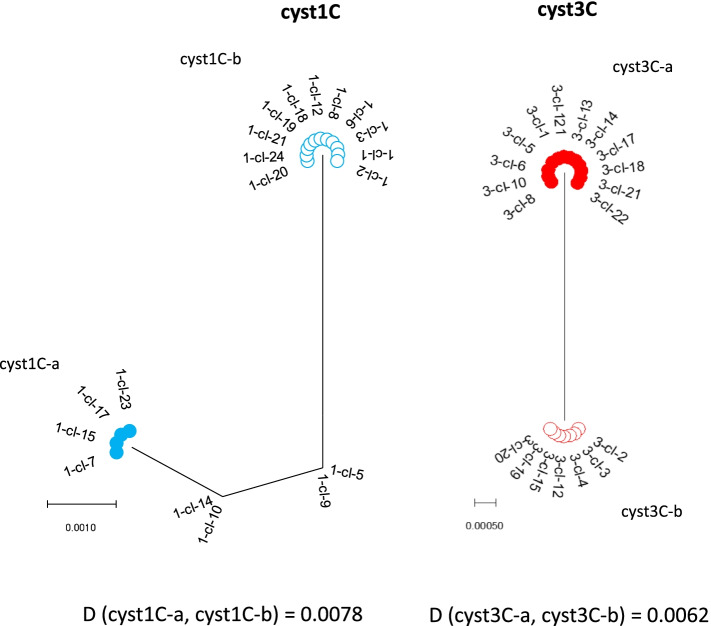
Phylogenetic analysis of *dis3* clones from cyst 1 and cyst 3 by maximum-likelihood method with gamma distribution. The analysis involved the nucleotide sequences. More than 2 identical clones within a single cyst are indicated as a cluster. The distance (D) between the clusters were computed with maximum composite likelihood model with gamma distribution

**Fig. 3:**
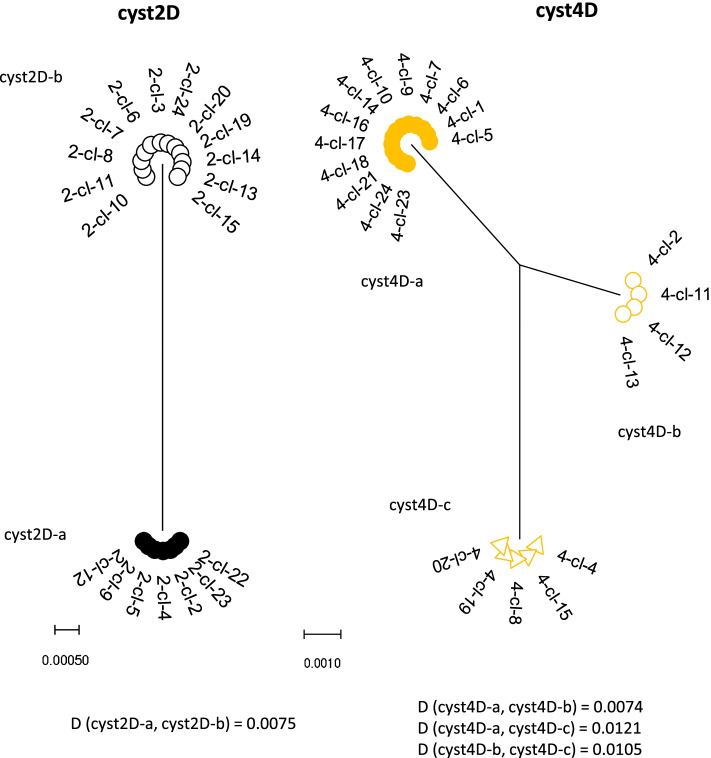
Phylogenetic analysis of *dis3* clones from cyst 2 and cyst 4 by maximum-likelihood method with gamma distribution. The analysis involved the nucleotide sequences. More than 2 identical clones within a single cyst are indicated as a cluster. The distance (D) between the clusters were computed with maximum composite likelihood model with gamma distribution

The phylogenetic analysis of the clones from the same single cyst is given in Figs. 2 and 3. The tree from cyst 1C consisted out of two haplotypes forming two clusters and two less common haplotypes in between. The expected haplotype distribution with four haplotypes of equal frequency did not yield a high probability for the observed data (*p *= 0.0092, Table S5), but the haplotypes with the lower frequency can be the result of recombination events between the haplotypes with the highest frequency (Figure S3). Phylogenetic analysis of cysts 2D and 3C demonstrated two haplotypes in each cyst, most likely representing the two non-daughter nuclei. The observed frequency is in agreement with a distribution of two haplotypes at equal frequency (*p* = 0.2513) or at 0.75 and 0.25 frequency (*p* = 0.2332), see Table S5. The tree from cyst 4D consists out of one haplotype with high frequency and two haplotypes of about half the frequency. The observed frequencies have the highest probability for a distribution of three haplotypes at a frequency of 0.5, 0.25 and 0.25 (*p* = 0.6643, Table S5). All the clones from cyst 50E were of the same haplotype (not shown), making the presence of more than one haplotype in the cyst very unlikely (*p* < 0.0001).

**Fig. 4:**
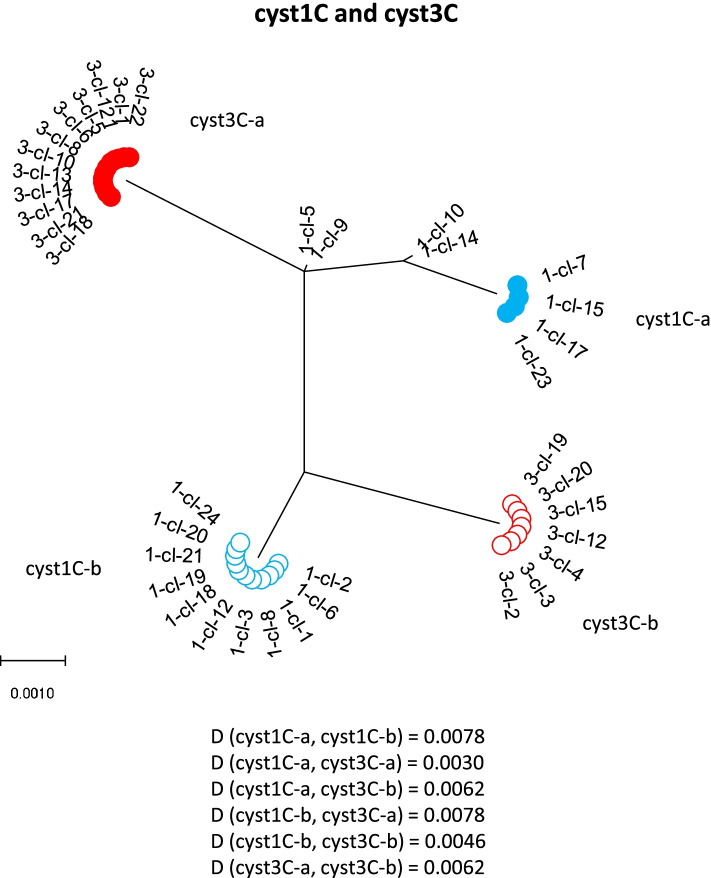
Phylogenetic analysis of the combined nucleotide sequences of *dis3* clones from cyst 1 and cyst 3 by maximum-likelihood method with gamma distribution. The analysis involved nucleotide sequences. More than 2 identical clones within a single cyst are indicated as a cluster. The distance (D) between the clusters were computed with maximum composite likelihood model with gamma distribution

**Fig. 5:**
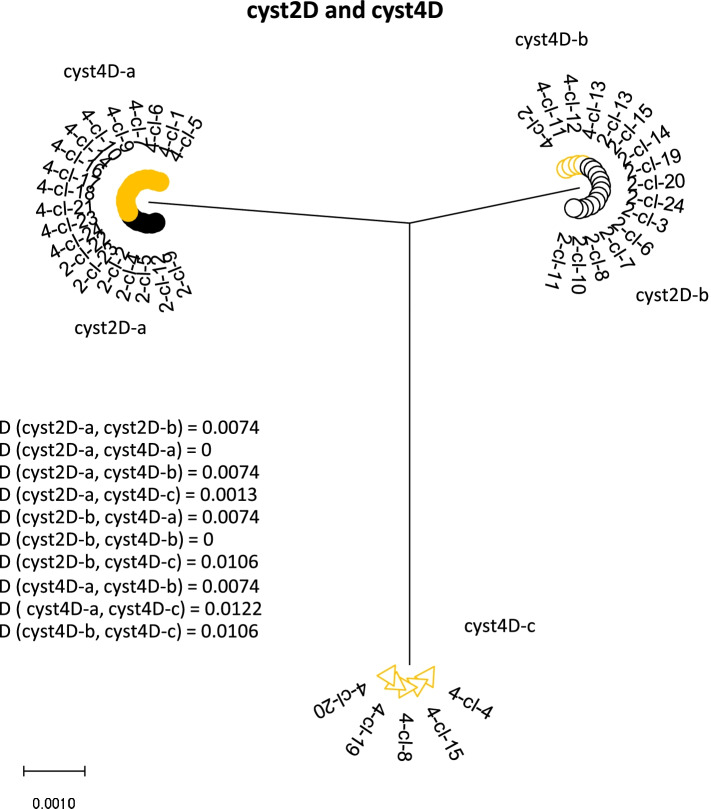
Molecular phylogenetic analysis of the combined nucleotide sequences of dis3 clones from cyst 2 and cyst 4 by maximum-likelihood method with gamma distribution. The analysis involved nucleotide sequences. More than 2 identical clones within a single cyst are indicated as a cluster. The distance (D) between the clusters were computed with maximum composite likelihood model with gamma distribution

Because the single cysts were all from the same dog [9], the phylogenetic relationship was also constructed for the combination of the two cysts of the same assemblage (Figs. 4 and 5). In the tree constructed from cysts 1C and 3C, six haplotypes can be found. The distance between the clusters from the putative daughter nuclei of the different cysts is smaller (0.0030 and 0.0046) than between the clusters from the non-daughter nuclei of the same cyst (0.0078 and 0.0062) or of the other cyst (0.0062 and 0.078). Cyst2D and 4D combined gave only three different haplotypes. Two haplotypes were found in both cysts, suggesting they represent the putative non-daughter nuclei of the two cysts. The third haplotype indicates heterozygosity within one of the pair of nuclei of cyst 4D.

### Discussion

The heterozygosity in the *gdh* and *dis3* fragments was measured in pooled cysts of assemblages with a variable level of ASH. The heterozygosity in *gdh* and *dis3* was found in concordance with the ASH of the whole genome of these assemblages: low in assemblages A and E, medium in assemblage B and high in assemblages C and D. We could not find any heterozygosity in the *gdh* and *dis3* fragments in assemblages A, E and F, which corresponds with the very low ASH in the genome of assemblage A and E (the ASH in the genome of assemblage F is not known) and it was demonstrated that the heterozygosity in assemblages C and D was, at least partly, caused by variation within single cysts. The assemblages A, E and F were identified as a monophyletic clade in the top of the rooted tree, as was found before [14].

Assemblage B showed much variation in the level of heterozygosity between the seven isolates, three isolates without heterozygosity and four isolates with between 0.21% and 0.62% variation. Woschke et al. (2021) found in assemblage B isolates in sequences of single copy genes in 59.1% of the sequences ambiguous nucleotides, very much in line with our data [20]. Lebbad et al., (2010) found out of five isolates of assemblage B, in one isolate ambiguous nucleotides in single copy genes [21]. The heterozygosity could be the result of differences between the cysts or within the cysts. In a study of Ankarklev et al. (2012) the variation in single cysts of assemblage B obtained from clinical samples the two loci Beta giardin (*bg*) and triosephosphate isomerase (*tpi*) were studied [11]. Heterozygosity (double peaks) was found within individual cysts in 21 out of the 36 cysts. Aligning all sequences from one patient did not create a consensus sequence, but subgroups were formed. Suggesting that patients were infected with mixed sub-assemblage infections. This is consistent with the diverse haplotypes of assemblage B found in a field study in Kenya [22] and explains the variation we found at pooled cysts level for the isolates of assemblage B. The assemblages C and D had the highest heterozygosity levels in pooled cysts. Gene duplication and/or aneuploidy [12, 23] as cause of the high heterozygosity is not likely, because the heterozygosity was seen in two different genes from different chromosomes, *gdh* from chromosome 4 and *dis3* from chromosome 5 [24].

High heterozygosity was also found within single cysts of assemblage C and D and confirmed the heterozygosity found in the genome wide analysis of the same cysts [9]. In the whole genome sequencing project ambiguous nucleotides were identified, but it was not possible to determine the ambiguous nucleotide distribution over the haplotypes. For the analysis of the haplotypes, it was crucial that all haplotypes in the cyst were amplified. Whole genome amplification by MDA prior to PCR is known to result in very low allelic sequence drop out. The completeness of the genomes (98.6–99.3% recovery of single copy genes) and therefore of the MDA products of which the genome was sequenced was demonstrated before [9]. The fact that cysts have four tetraploid nuclei, contributes most likely also to the completeness of the genome in the MDA products. Because the single copy genes will have four copies of each of the four haplotypes in a single cyst, the use of a single cyst also decreased the chance alleles are missed, compared to the use of single trophozoites. More than four haplotypes of a single copy gene in a cyst can only happen when that gene mutates at a very high rate during the encystation. Especially, in our case with a relatively small gene fragment of *dis3*, it is very unlikely.

In the *dis3* clones, singleton sequences were found. In the four cysts of assemblage C and D, in total 12 singleton sequences were identified with on seven positions an alternative nucleotide in only one clone. In five cases the alternative nucleotide was not found in the whole genome sequence of the same cysts. This suggests that these five alternative nucleotides were introduced by PCR, cloning and/or Sanger sequencing after the MDA production. For the other two cases the frequency of the alternative nucleotide in the whole genome sequences was low (14.9% and 9.1%). Because, by definition, the percentage of alternative nucleotides has to be higher than 15% [6], these two cases do not contribute to the ASH, although one was very close to the breach. Because whole genome sequencing and cloning were performed on the same MDA product, the two positions with alternative nucleotides could be from natural origin, but could also be introduced by MDA, despite the high fidelity of the Φ29 polymerase used in MDA [16, 25, 26]. The single cyst 50E from assemblage E from isolate 50 with no heterozygosity in the pooled cysts had the same number of singletons as the other cysts. This indicates that despite of our attempts to reduce artefacts, we were not able to prevent them all. However, the fact that after removal of the singleton sequences, all ambiguous positions in the whole genome sequences that contribute to the ASH were still represented by the clones justified the removal of these singletons.

In the four single cysts from assemblage C and D we found two to four haplotypes per cyst. In cysts 2D and 3C, only two haplotypes per cyst were found. Assuming the differences between the non-daughter nuclei to be greater than between the daughter nuclei, these two haplotypes represent the haplotypes from the non-daughter nuclei within the cyst. The heterozygosity at nucleotide level of 0.60% and 0.75% was comparable with the ASH in the whole genome of those assemblages. In cyst 4D three haplotypes were found and based on the frequency, the most likely distribution is that the most abundant haplotype represents the homozygous pair of nuclei, whereas the two haplotypes of about half the frequency represents the heterozygous non-daughter pair of nuclei. With three haplotypes and only two types of nuclei (pair of non-daughter nuclei), there must be heterozygosity within the same type of nuclei anyhow. The haplotypes from cyst 4D differ in five nucleotides or more and all haplotypes were found in at least four clones, making artefacts unlikely. The distribution of the four haplotypes from cyst 1C is less clear. Four haplotypes with the same frequency from two heterozygous nuclei is unlikely, because of the very different frequencies of the haplotypes (different numbers of clones). Recombination, natural or an artefact, of the two haplotypes with the highest frequency as parent sequences can result in the two haplotypes with lower frequency. If it is not an artefact, the recombination must have occurred between non-daughter nuclei. Another option is aneuploidy. Tůmová et al. (2016) have found aneuploidy in several isolates, all from assemblage AI, resulting in duplication of otherwise single copy genes [23]. However, it is not known whether aneuploidy also occurs in other assemblages than AI.

Comparing the two cysts from the same assemblage with each other, it was remarkable that the haplotypes from cyst 2D were identical with two of the three haplotypes from cyst 4D, demonstrating, in this case, that the non-daughter nuclei within the same cyst are more divers than the putative daughter nuclei from two different cysts of the same host. In the cysts from assemblage C, the distances between the haplotypes from the putative daughter nuclei were also smaller than the distances between the non-daughter nuclei of the same cyst, but they were not identical as in cysts 2D and 4D. This can be related to the assemblage, but it can also be related to the origin of these cysts. Although the cysts 1C and 3C were from the same dog, they do not necessarily have the same predecessor and when they have, the number of duplications of the trophozoite before the encystation can be much larger than in the case of cyst 2D and 4D. Finding only one haplotype in cyst 50E from assemblage E agreed with the absence of heterozygosity in the pooled cysts from the same isolate. Disintegration of the cyst and/or DNA is not likely as explanation for finding only one haplotype, because in the 10,000 pooled cyst of the same isolate, there was also only one haplotype found. Furthermore, cyst 50E was treated as the cysts from assemblage C and D and only discrete, intact nuclei were detected after labeling and the single peak in DAPI signal during sorting also indicated equal amounts of DNA in all cysts. We did not found four nuclei in all cysts, but about six % of the cysts had only three nuclei. Poxleitner et al., (2008) found in 19% of viable cysts three nuclei, presumably, because of fusion of two nuclei and not because of disintegration [12]. We only cloned in total five cysts, but because the results closely matched with the whole genome sequences (assemblages C and D) and with the results obtained from the pooled cysts of the same assemblages (assemblages C, D and E), we are convinced the results are valid. However, variation between cysts of the same isolate can occur and indeed have been found even within this small number of cysts used in this study. Another implication of this is that the level of heterozygosity determined from pooled cysts or trophozoites can differ from that which is obtained from single cysts. The heterozygosity can be underestimated, because the variation within the individual cysts can be diluted by the other cysts to below the threshold. Conversely, the heterozygosity can be an overestimation, because the variation of the individual cysts was added up. We could find only two other publications where they described the variation within *Giardia* cysts and/or trophozoites. Aguilar et al., (2015) described the variation within 10 cysts from a human isolate [10]. Nine cysts of assemblage AII were without variation, while one cyst was of mixed AII-B assemblage. Cloning of PCR products from that cyst resulted in 14 alleles of the *bg* gene and 9 alleles of the orfc4 gene. Because of the high numbers of alleles and because of the single cyst seemed to have sequences of both assemblage A and B, contamination and/or artefacts cannot be excluded completely. This was also admitted by the authors. Ankarklev et al., (2012) studied single cysts and trophozoites of assemblage B from human isolates [11]. They also found variation within individual trophozoites and cyst in fragments of the *tpi* and *bg* gene. However, they did not clone the PCR products with the ambiguous nucleotides and therefore the number of haplotypes per cell could not be determined.

The absence of variation demonstrated in single cyst of assemblage E and in pooled cysts of assemblages A, E and F, indicates that the cysts from this clade are homozygous, whereas the cysts from assemblages C and D are heterozygous. Even in the relatively small fragments of the *gdh* and *dis3* fragments, located on different chromosomes, variation in nucleotide and amino acid sequences could be demonstrated. Advantages of being a heterozygous polyploid in general can be found in heterosis and gene redundancy [27]. These effects mean that heterozygotes have a higher fitness than homozygotes. It makes the duplicated copy of an essential gene available for “evolutionary experiments” and diversifying gene functions [27]. Increased heterozygosity in *Candida* is related with increased drug resistance [28] and also for *Giardia* it is suggested that the high ASH in assemblage B favors drug resistance compared to assemblage A with much lower ASH [29]. The advantage of heterozygosity combined with polyploidy has been suggested before, because a polyploid *Giardia* with a malfunctioning allele can survive, leaving time for that allele to develop into a new function [22]. One of the disadvantages of creating heterozygosity by becoming polyploid can be the increased size of the nucleus, necessary to contain the increased DNA contents [27]. However, this is not valid for *Giardia*, because *Giardia* is a polyploid organism by itself, not only the heterozygotes and the DNA content is divided over two nuclei. However, based on the phylogenetic analysis, the clade with assemblages A, E and F with low ASH originated from predecessors with most likely a high ASH. This suggests that having a low ASH must have advantages as well. Having a low ASH can therefore be considered as a derived characteristic; the ability to have a high heterozygosity is lost and the ability to have a low heterozygosity is acquired.

The low ASH in some assemblages is suspected to be the results of DNA exchange between the non-daughter nuclei followed by homologous recombination [3, 12]. Because the assemblages with low ASH (assemblages A, E and F) form one clade in the top of the rooted tree [14], increased homologous recombination is expected to be a characteristic developed during the evolution of *Giardia*. Previous research has been focused on assemblage AI (isolate WB). It was demonstrated that episomal DNA present in only one nucleus of a trophozoite, remains in one nucleus after propagation of the trophozoites, but appeared in up to three nuclei after encystation and subsequently excystation by fusion of the non-daughter nuclei and DNA exchange, named diplomixis [12]. Later this was also demonstrated for chromosomal genetic material [3]. The evidence of diplomixis was strengthened by the fusion of the nuclear envelopes seen between the nuclei within a single Giardia cyst [30]. Nuclear sorting (all daughter cells receive two identical daughter nuclei) as homogenization mechanism seems not to happen in *Giardia* [3], making the exchange of DNA between non-daughter nuclei followed by homologous recombination the most likely mechanism to reduce ASH. Most of this was demonstrated in axenic cultures of assemblage AI, an assemblage with low ASH and therefore, most likely high capacity for recombination. The assemblages C and D cannot be cultured yet, but because of the high ASH it would be interesting to study diplomixis (or the absence of it) and the genes active during encystation in the assemblages C and D as well.

Being an early branch of eukaryotes, *G. duodenalis* is known to lack certain genes involved in processes of DNA repair[31], possibly resulting in higher chances of replication errors slipping through. Meiosis is never observed in *Giardia* spp., nevertheless, sequence analysis revealed several homologs of meiosis-specific genes (HMG’s): Hop1, Spo11, Dmc1a, Dmc1b and Mnd1 [12]. Possibly, these genes have slightly different functions in *Giardia* and it is hypothesized that genes involved in meiosis originated from genes involved in DNA repair [32]. Despite the absence of meiosis, Dmc1a, Spo11 and Hop1 were found to be expressed during encystation, the stage in which diplomixis took place [3]. Therefore, these genes deserve extra attention in the study on homologous recombination.

### Conclusions

We found the levels of heterozygosity in *gdh* and *dis3* for the assemblages A to F to be related to the ASH. No heterozygosity was found in the clade formed by the assemblages A, E and F, in contrast to the assemblages C and D at the root of the tree with a high ASH. This confirmed the hypothesis that having high heterozygosity may be a primitive character, whereas having low heterozygosity is a derived character. Analysis of the haplotypes within individual cysts showed the fundamental difference in organization of the genetic material between assemblages with a high and a low ASH. In single cysts with two haplotypes, the haplotypes represent most likely the two non-daughter nuclei. Three or four haplotypes within single cysts can only be explained by assuming heterozygosity also within the daughter nuclei. Presence of only one haplotype within a single cyst of assemblage E confirmed the absence or very low level of variation in assemblage E. Differences in double-strand DNA repair and/or recombination are expected between the clades with low ASH (A, E, F) and high ASH (B, C and D).

## Supplementary Information

**Additional file 1: Table S1.** Origin of samples and date of entry at Veterinary Microbiological Diagnostic Centre (VMDC). **Table S2.** PCR Primers and amplicon size of the product. **Table S3.** Probability P for detecting haplotypes in different numbers of clones. **Table S4.** Ambigous nucleotides in cloned dis3 fragments from single cysts. The singleton sequences are given in red and are considered artefacts. **Table S5.** Probability of the observed # clones per haplotype (H1, H2, H3, H4) to be in agreement with the expected frequency of haplotypes. This was performed for 1 to 4 expected haplotypes. All combinations (*n*=5) of expected haplotypes are given. When observed number of haplotypes are higher than expected number of haplotypes, *p*=0.

**Additional file 2: Figure S1.** Phylogenetic analysis by maximum likelihood method of dis3 fragments amplified from pooled cysts of assemblages A to F. Samples with the assemblage and the number of the isolate (green squares) and reference sequences obtained from genome sequence projects (red dots) are indicated. Bootstrap values are indicated at the branching points. Bar indicates number of nucleotide substitutions per site. **Figure S2.** Isolate 50 (assemblage E) sorted by FACS. A) Gate set for Giardia cysts (black circle) with DAPI signal on x-axis and FITC signal on y-axis. Particles in left corner were added E. coli. B) Histogram of DAPI signal with intensity (x-axis) and particle counts (y-axis). C) Cysts labeled with FITC (cyst wall protein in green) and DAPI (nuclei in blue). Photo was taken by fluorescent microscopy. **Figure S3.** Possible recombination events in dis3 fragment of cyst 1C.

## Data Availability

All sequence data are submitted to Genbank (accession numbers ON360029-ON360071). Other data not provided as supplementary data are available from the corresponding author on reasonable request.
